# Bacterial endotoxin enhances colorectal cancer cell adhesion and invasion through TLR-4 and NF-*κ*B-dependent activation of the urokinase plasminogen activator system

**DOI:** 10.1038/sj.bjc.6604942

**Published:** 2009-05-12

**Authors:** S D Killeen, J H Wang, E J Andrews, H P Redmond

**Affiliations:** 1 Department of Academic Surgery, Cork University Hospital and University College Cork, Cork, Ireland

**Keywords:** perioperative metastatic tumour growth, lipopolysaccharide, extracellular matrix, urokinase plasminogen activator system, Toll-like receptor 4

## Abstract

Perioperative exposure to lipopolysaccharide (LPS) is associated with accelerated metastatic colorectal tumour growth. LPS directly affects cells through Toll-like receptor 4 (TLR-4) and the transcription factor NF-*κ*B. The urokinase plasminogen activator (u-PA) system is intimately implicated in tumour cell extracellular matrix (ECM) interactions fundamental to tumour progression. Thus we sought to determine if LPS directly induces accelerated tumour cell ECM adhesion and invasion through activation of the u-PA system and to elucidate the cellular pathways involved. Human colorectal tumour cell lines were stimulated with LPS. u-PA concentration, u-PA activity, active u-PA, surface urokinase plasminogen activator receptor (u-PAR) and TLR-4 expression were assessed by ELISA, colorimetric assay, western blot analysis and flow cytometry respectively. *In vitro* tumour cell vitronectin adhesion and ECM invasion were analysed by vitronectin adhesion assay and ECM invasion chambers. u-PA and u-PAR function was inhibited with anti u-PA antibodies or the selective u-PA inhibitors amiloride or WXC-340, TLR-4 by TLR-4-blocking antibodies and NF-*κ*B by the selective NF-*κ*B inhibitor SN-50. LPS upregulates u-PA and u-PAR in a dose-dependent manner, enhancing *in vitro* tumour cell vitronectin adhesion and ECM invasion by >40% (*P*<0.01). These effects were ameliorated by u-PA and u-PAR inhibition. LPS activates NF-*κ*B through TLR-4. TLR-4 and NF-*κ*B inhibition ameliorated LPS-enhanced u-PA and u-PAR expression, tumour cell vitronectin adhesion and ECM invasion. LPS promotes tumour cell ECM adhesion and invasion through activation of the u-PA system in a TLR-4- and NF-*κ*B-dependent manner.

Surgery remains the only definitive curative modality for colorectal cancer (Meyerhardt and Mayer, 2005). Unfortunately, the perioperative milieu may facilitate the progression of previously dormant occult metastatic disease (Da Costa *et al*, 1998). Although the mechanisms underlying this phenomenon are not fully elucidated, implicated factors include perioperative immune suppression (Li *et al*, 2001), elevated angiogenic and inflammatory cytokine levels (Da Costa *et al*, 1998) and elimination of putative tumour-suppressing agents with primary tumour excision (Coussens and Werb, 2002).

Lipopolysaccharide (LPS) or endotoxin, the foremost glycolipid outer membrane constituent of Gram-negative bacteria, potently stimulates immune system cells by binding cell-surface Toll-like receptor 4 (TLR-4) and activating transcription factors and protein kinases, such as NF-*κ*B and p38 kinase, resulting in an increased production of pro-inflammatory cytokines, overexpression of cell adhesion molecules and matrix-degrading enzymes (Manthey *et al*, 1992). Contemporary studies have demonstrated the expression of TLR-4 on human colorectal cancer cells and highlighted a key function for the TLR system in the development of colitis-associated tumours, suggesting a role for this receptor in colorectal cancer development and progression (Böcker *et al*, 2003; Fukata *et al*, 2007).

LPS has recently been implicated in accelerated metastatic tumour growth after surgery. Following laparotomy and air laparoscopy, LPS contaminates the peritoneal cavity and enters the systemic circulation due to perioperative bacterial gut translocation. In murine metastatic breast carcinoma models, mice undergoing laparotomy or air laparoscopy had increased serum levels of LPS and metastatic tumour burden compared to those in the CO_2_ laparoscopy group, a result replicated by intraperitoneal LPS injection (Pidgeon *et al*, 1999; Harmey *et al*, 2002). This association between LPS and accelerated perioperative tumour growth may be a manifestation of a direct effect on cancer cells or a circuitous immunologically mediated singularity. Luo *et al* (2004) suggest that endotoxin is subservient to inflammatory cytokines produced primarily by the innate immune system, as the LPS-induced enhanced tumour burden was TNF-*α* dependant and abolished in TLR4-deficient mice.

Although it has been extensively demonstrated that bacterial endotoxin (LPS), analysed in both *in vitro* and *in vivo* experimental settings, reduces apoptosis (Andrews *et al*, 2001; Wang *et al*, 2003) and increases proliferation in metastatic tumour cells (Coffey *et al*, 2006), the role of extracellular matrix (ECM)-degrading enzyme systems remains to be elucidated in this phenomenon.

For a tumour to become invasive and ultimately metastasise, tumour cells must cross cellular and matrix boundaries by attaching to, interacting with, and invading components of the basement membrane and ECM, culminating in access to the circulation (Hart and Saini, 1992; Al-Mehdi *et al*, 2000). Comprising several interdependent components, the urokinase plasminogen activator (u-PA) system has a number of distinct but complementary functions in this tightly regulated multi-step process. The serine protease u-PA is secreted as a 55 kDa proenzyme proteolytically cleaved to an enzymatically active two-chain form (Pollanen *et al*, 1988; Sidenius and Blasi, 2003). Its 60 kDa glycosylphosphatidylinositol-linked receptor, urokinase plasminogen activator receptor (u-PAR/CD87), binds the epidermal growth factor-like domain of active u-PA (Vassalli *et al*, 1985; Pollanen *et al*, 1990) and the ECM constituent vitronectin (Roldan *et al*, 1990; Waltz and Chapman, 1994). Plasminogen activator inhibitors 1 (PAI-1; Kanse *et al*, 1996) and PAI-2 form trimeric complexes with u-PAR bound u-PA, culminating in internalisation, u-PA/PAI degradation and u-PAR recycling (Declerck *et al*, 1988). u-PA/u-PAR promotes cell/cell and cell/ECM proteolysis by regulating localised plasminogen activation (Cubellis *et al*, 1990). Independent of proteolysis, u-PA enhances cell invasion through activation of several migration-associated signalling molecules such as extracellular signal-regulated kinases (Nguyen *et al*, 1998), focal adhesion kinases (Tang *et al*, 1998) and signal transducers and activators of transcription 1 (STAT1) (Dumler *et al*, 1998). Such intercellular signal transduction is apparently facilitated by the interaction of u-PAR with integrins (Wei *et al*, 1996; Degryse *et al*, 2001) and cytoskeletal components (Xue *et al*, 1997). In addition, u-PA/u-PAR mediates cellular adhesion to the ECM protein, vitronectin directly through integrin-independent, high-affinity interaction between u-PAR and vitronectin, and indirectly through function modifying lateral associations with integrin family members (Xue *et al*, 1997; Degryse *et al*, 2001).

A number of experimental and clinical studies highlight the significance of the u-PA system in colorectal cancer abound (Ertongur *et al*, 2004; Setyono-Han *et al*, 2005). Both Harvey *et al* (1999) and Skelly *et al* (1997) demonstrated superior 5-year survival rates in patients whose tumour had lower total u-PA expression after curative colon cancer resection. Herszényi *et al* (2008) showed elevated serum levels of u-PA in patients with colorectal cancer. A high u-PAR concentration in resected colorectal cancers is an independent and significant prognostic factor for 5-year overall survival (Ganesh *et al*, 1994). It has also been demonstrated that a serum protein fraction representing soluble u-PAR (su-PAR: u-PAR protein without the glycolipid anchor) is inversely correlated with survival (Stephens *et al*, 1999). Suzuki *et al* (1998) showed that u-PAR expression increases during the transition from adenoma to invasive carcinoma in colorectal epithelium.

Despite being implicated in promoting colon cancer progression, the effect of LPS on u-PA and u-PAR expression, and the function of this system in endotoxin augmented colon cancer cell invasiveness, is not known. The aim of this study, therefore, was to determine if the u-PA system is involved in endotoxin-enhanced tumour cell adhesion and extracellular invasion, and to elucidate the function of TLR-4 and NF-*κ*B in this process.

## Materials and methods

### Reagents and antibodies (Abs)

Medium L-15, DMEM, HBSS, PBS without Ca^2+^ and Mg^2+^, fetal calf serum (FCS), penicillin, streptomycin sulphate, glutamine and 0.05% trypsin/0.02% EDTA solution were purchased from Invitrogen Life Technologies (Paisley, Scotland, UK). Human vitronectin, LPS (*Escherichia coli* O55B5), and all other chemicals unless indicated were from Sigma-Aldrich (St Louis, MO, USA). Human u-PA, the cell-permeable NF-*κ*B inhibitor peptide, SN-50 and its non-functioning control analogue SN-50M were obtained from Calbiochem (San Diego, CA, USA). Monoclonal anti-u-PA and anti-u-PAR Abs were obtained from Santa Cruz Biotechnology (Santa Cruz, CA, USA). Anti-TLR-4-blocking mAb (clone, HTA-125), FITC-conjugated anti-TLR-4 mAb and isotype-control Abs were from eBioscience (San Diego, CA, USA) and Serotec (Oxford, UK) respectively. Anti-u-PAR (CD87) mAb and its isotype-control mAb were from R&D systems (Minneapolis, MN, USA). Horseradish-peroxidase-conjugated secondary mAbs were purchased from Dako (Cambridgeshire, UK). The novel u-PA inhibitor, WXC-340, was kindly donated by Dr Bernd Muehlenweg (Wilex AG, Munich, Germany).

### Cell culture

Human colorectal tumour cell lines SW480, SW620 and CACO2 were obtained from American Type Culture Collection (Manassas, VA, USA). SW480 and SW620 cells were grown in medium L-15 whereas CACO2 cells were cultured in DMEM. Culture medium was supplemented with 10% FCS, penicillin (100 U ml^−1^), streptomycin sulphate (100 *μ*g ml^−1^) and glutamine (2.0 mM). Cells were maintained at 37°C in a humidified 5% CO_2_ atmosphere and subcultured by trypsinisation with 0.05% trypsin/0.02% EDTA when cells became confluent. All studies were performed within 10 passages of obtaining the cell lines.

### Cell stimulation and sample preparation

Cells cultured in six-well plates (1 × 10^6^ cells per well; Falcon, Lincoln Park, NJ, USA) were exposed to various concentrations of LPS (0.1, 1 and 10 *μ*g ml^−1^) for different time periods (0, 6, 12, 18 and 24 h) at 37°C in humidified 5% CO_2_ conditions. Cell-free supernatants were collected by centrifugation at 400 **g** for 10 min and frozen at −70°C or analysed immediately. To assess the function of protein synthesis in LPS-enhanced u-PA and u-PAR expression, cells were co-incubated with various concentrations of cycloheximide (Paisley, UK).

Western blot analysis for TLR-4 involved unstimulated cell lysates. To examine the role of TLR-4 and NF-*κ*B in LPS-enhanced u-PA and u-PAR expression, cells were incubated with various concentrations of TLR-4-blocking Ab and SN-50, respectively, before LPS stimulation. In dose–response experiments the optimal inhibitory concentration was determined and used in all subsequent experiments (25 and 100 *μ*g ml^−1^ respectively). CACO2 cells were cultured with 1 mM butyrate for 24 h as a positive control as described by Gibson *et al* (1999).

For western blot analysis of cell supernatant u-PA, conditioned medium was concentrated 90- to 100-fold using centricon 10 centrifugal filter units (Millipore, Bedford, MA, USA) with a 10 kDa pore diameter cutoff. Protein concentrations were determined using a Micro BCA protein assay reagent kit (Pierce, Rockford, IL, USA). Cell homogenate total protein samples were mixed loading buffer (60 mM Tris, 2.5% SDS, 10% glycerol, 5% mercaptoethanol, 0.01% bromophenol blue) in a 1 : 1 ratio whereas concentrated conditioned medium for cell supernatant u-PA western blot analysis was mixed with sample buffer 3 : 1 ratio. Samples were denatured for 10 min at 100°C.

### u-PA and u-PAR ELISA

Levels of u-PA and PAI-1 in culture supernatants and cell homogenates, and u-PAR in cell homogenates, were measured using commercially available u-PA and u-PAR ELISA kits (American Diagnostica, Greenwich, CT, USA) according to the manufacturer's instruction.

### UPA activity assay

The urokinase plasminogen activator activity levels in cell supernatants were measured using a commercially available chromogenic u-PA activity assay kit (Chemicon, Temecula, CA, USA). Values are expressed as IU per mg protein.

### Western blot analysis

Aliquots containing equal amount of total proteins from each sample were separated in SDS-polyacrylamide gels and electrophoretically transferred onto nitrocellulose membranes (Schleicher & Schuell, Dassel, Germany). Membranes were blocked for 1 h at room temperature with PBS containing 0.05% Tween 20 and 5% non-fat milk, and probed overnight at 4°C with primary Abs at conditions recommended by the manufacturers. Blots were washed three times with PBS containing 0.05% Tween 20 and 5% non-fat milk and further incubated with the appropriate horseradish-peroxidase-conjugated secondary Ab at room temperature for 1 h. Immunoreactive proteins visualised using the ECL detection system (Amersham Biosciences, Piscataway, NJ, USA). To ensure equal protein loading, all membranes were stripped and re-probed with anti-*β*-actin Ab where indicated. Western blot analysis studies were performed in duplicate and repeated on three separate occasions.

### Measurement of NF-*κ*B activation

SW480, SW620 and CACO2 cells were incubated with 0.1 *μ*g ml^−1^ LPS for 30 min. Briefly, cells were lysed in a hypotonic solution (10 mM HEPES, 1.5 mM MgCl_2_, 10 mM KCl and 0.1% Nonidet P-40, pH 7.9) on ice for 10 min and centrifuged at 13 000 r.p.m. to pellet nuclei. Cytoplasmic supernatants were removed, and nuclei were re-suspended in nuclear extract buffer (20 mM HEPES, 25% glycerol, 420 mM NaCl, 1.5 mM MgCl_2_ and 0.2 mM EDTA, pH 8.0) on ice for 15 min. The lysates were centrifuged at 13 000 r.p.m., and supernatants containing the nuclear proteins were collected. All buffers contained freshly added 0.5 mM DTT, 0.5 mM PMSF and protease inhibitor cocktail (Roche, Mannheim, Germany). Protein concentrations were determined using a Micro BCA protein assay reagent kit (Pierce). All extracts were stored at −70°C until analysed. NF-*κ*B activation was measured by the NF-*κ*B ELISA kit (Active Motif, Carlsbad, CA, USA) according to the manufacturer's recommendations.

### FACS analysis

Cells were exposed to various concentrations of LPS (0.1, 1 and 10 *μ*g ml^−1^) for different time periods (0, 6, 12, 18 and 24 h) at 37°C in humidified 5% CO_2_. The expression of u-PAR and TLR-4 on SW480, SW620 and CACO2 cells was determined using direct immunofluorescent staining. Briefly, 20 *μ*l of FITC-conjugated anti-u-PAR or FITC-conjugated anti-TLR-4 mAbs was added to 100 *μ*l of cell suspension (1 × 10^6^ cells per ml) and incubated at 4°C for 30 min. FITC-conjugated isotype IgG1 mAbs were used as negative controls. Cell-surface expression of u-PAR and TLR-4 was analysed on a FACScan flow cytometer (BD Biosciences, Mountain View, CA, USA) to detect the log of the mean channel fluorescence intensity with an acquisition of FL1. A minimum of 10 000 events were collected and analysed on CellQuest software (BD Biosciences).

### Tumour cell vitronectin adhesion assay

Human vitronectin (1 *μ*g ml^−1^), poly-Lysine (1 *μ*g ml^−1^) and BSA (1 *μ*g ml^−1^, used as a negative control) were coated onto 96-well, flat-bottom plates (Falcon). SW480, SW620 and CACO2 cells were exposed to various concentrations of LPS (0.1, 1 and 10 *μ*g ml^−1^) for different time periods (0, 6, 12, 18 and 24 h) at 37°C in humidified 5% CO_2_ conditions. For blocking experiments, cells were pre-treated with various concentrations of SN-50 (100 *μ*g ml^−1^), SN-50M (100 *μ*g ml^−1^), u-PA inhibitors (10 mg ml^−1^ amiloride, 0.3 mg ml^−1^ WXC-340), u-PA (20 *μ*g ml^−1^), u-PAR (20 *μ*g ml^−1^) and TLR-4-blocking Abs (25 *μ*g ml^−1^) for 1 h before LPS stimulation. The above cell suspension (100 *μ*l; 1 × 10^6^ cells per ml) was added in triplicate to the vitronectin-, poly-Lysine- and BSA-coated 96-well plates and incubated for 2 h at 37°C in humidified 5% CO_2_ conditions. The cell suspension was discarded and the remaining adherent cells were washed twice with PBS. Fluorescent probe precursor (100 *μ*l), calcein-AM (Calbiochem) was added to each well. Fluorescence was measured using a fluorescence plate reader at an excitation wavelength of 485 nm and emission wavelength of 520 nm. Standard curves to convert measured fluorescence to cell number were constructed utilising known cell numbers.

### Tumour cell invasion assay

*In vitro* tumour cell invasion was assessed using an ECM *in vitro* ECM invasion chambers (Chemicon) with cell culture inserts containing an 8*μ*m pore size positron emission tomography membrane with a thin layer of ECM membrane matrix as previously described. Briefly, 0.5 ml of tumour cells (1 × 10^5^ cells per ml) re-suspended in serum-free medium containing various concentrations of LPS (0.1, 1 or 10 *μ*g ml^−1^) was added to the cell culture insert of the invasion chamber. FBS (20 *μ*g ml^−1^) was added in the outer chamber as a chemoattractant. SW480 and SW620 cells were pretreated with various concentrations of SN-50 (100 *μ*g ml^−1^), SN-50M (100 *μ*g ml^−1^), u-PA inhibitors (10 mg ml^−1^ amiloride, 0.3 mg ml^−1^ WXC-340), u-PA (20 *μ*g ml^−1^), u-PAR (20 *μ*g ml^−1^) and TLR-4-blocking Abs (25 *μ*g ml^−1^) for 1 h before LPS stimulation. The cells were then incubated at 37°C in humidified 5% CO_2_ conditions for 24 h. Invaded cells that attached to the bottom of the matrix membrane were detached and lysed in cell lysis buffer. Cell lysates were then stained with CyQuant-GR-Dye (Chemicon). Fluorescence was measured using a fluorescence plate reader at an excitation wavelength of 485 nm and emission wavelength of 520 nm. A standard curve to convert measured fluorescence to cell number was constructed using known cell numbers.

### Statistical analysis

All data are presented as the mean±s.d. Student's two-tailed *t*-test was used to compare data between two groups. One-way analysis of variance and Bonferroni's correction were used to compare data between three or more groups. Differences were judged statistically significant at *P*<0.05.

## Results

### LPS enhances u-PA release and activity in colon cancer cells

**Figure 1:**
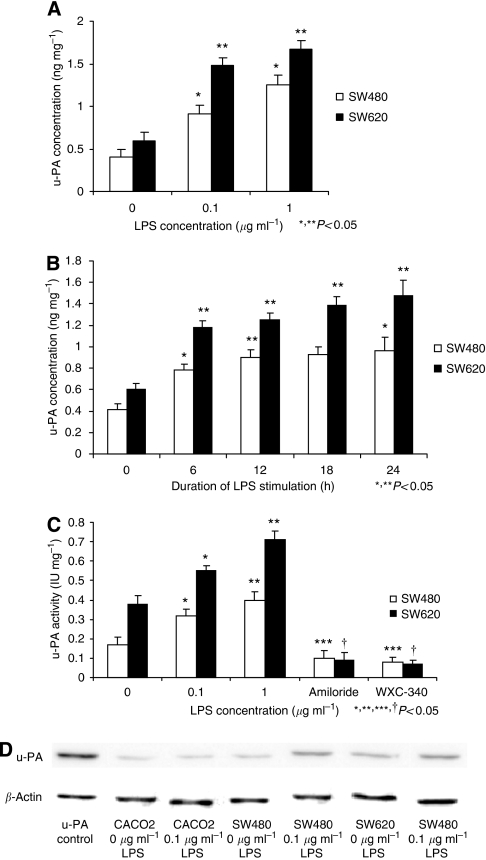
(**A**) LPS-stimulated tumour cells release u-PA in a dose-dependent manner. Following stimulation with various concentrations of LPS for 24 h, u-PA concentration in SW480 and SW620 cell culture supernatants was measured by ELISA as described in Materials and Methods. Data are expressed as the mean±s.d. (ng mg^−1^ of protein) of three separate experiments conducted in triplicate. The statistical significance was compared with the cells incubated with culture medium (^*^ for 0.1 *μ*g ml^−1^, *P*<0.05) and for cells incubated with 0.1 *μ*g LPS (^**^P⩽0.05 for 1 *μ*g LPS compared to 0.1 *μ*g LPS). (**B**) LPS statistically increases u-PA release within 12 h of stimulation. Following stimulation with 0.1 *μ*g ml^−1^ LPS for various time periods, u-PA concentration in SW480 and SW620 cell culture supernatants was measured by ELISA as described in Materials and Methods. Data are expressed as the mean±s.d. (ng per mg of protein) of three separate experiments conducted in triplicate. The statistical significance was compared with the cells incubated with culture medium (^*^ for 0.1 *μ*g ml^−1^, ^**^ for 1 *μ*g ml^−1^, *P*<0.05). (**C**) LPS enhances u-PA activity in SW480 and SW620 cell supernatants. SW480 and SW620 cells were stimulated with various concentrations of LPS for 24 h in the presence or absence of the selective u-PA inhibitors amiloride (10 mg ml^−1^) and WX-340 (1.0 mg ml^−1^). The u-PA activity in culture supernatants was measured by colorimetric analysis as described in Materials and Methods. Data are expressed as the mean±s.d. (IU per mg of protein) from three separate experiments conducted in triplicate. The statistical significance was compared with the cells incubated with culture medium alone (^*^ for 0.1 *μ*g ml^−1^, ^**^ for 1 *μ*g ml^−1^, *P*<0.05). (**D**) LPS increases active u-PA expression in SW480 and SW620 cell supernatants. Following stimulation with LPS for 24 h, tumour cell culture supernatants were processed and concentrated as described in Materials and methods and western blot analysis was performed to detect active u-PA expression. Lane 1, u-PA positive control; lanes 2 and 3, CACO2 cells stimulated with 0 and 0.1 *μ*g ml^−1^ LPS respectively; lanes 4 and 5, SW480 cells stimulated with 0 and 0.1 *μ*g ml^−1^ LPS respectively; lanes 6 and 7, SW620 cells stimulated with 0 and 0.1 *μ*g ml^−1^ LPS respectively.

Stimulation of SW480 and SW620 tumour cells with LPS increased u-PA protein release in a dose-dependent manner. LPS at 0.1 *μ*g ml^−1^ was sufficient to enhance u-PA levels in the cell supernatants (Figure 1A). This occurred within 12 h of LPS stimulation (Figure 1B). LPS stimulation induced increased u-PA activity in both SW480 and SW620 cell supernatants (Figure 1C). This was mirrored by increased expression of activated two-chain u-PA in SW480 and SW620 cells following LPS stimulation on western blot analysis of concentrated cell supernatants (Figure 1D). Nevertheless, LPS-stimulated upregulation of u-PA activity was almost completely blocked by two u-PA selective antagonists, amiloride and WXC-340 (Figure 1C). Metastatic SW620 cells had higher baseline u-PA protein and activity levels than primary SW480 cells (Figure 1A and C).

### LPS enhances u-PAR expression in colon cancer cells

**Figure 2:**
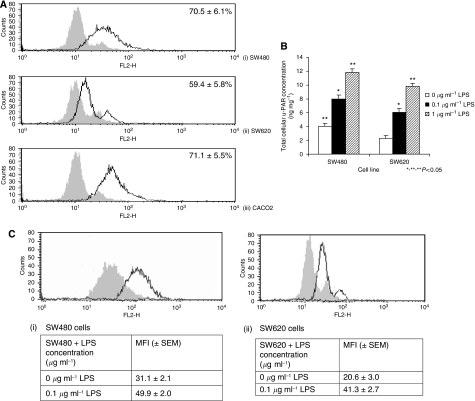
(**A**) SW480, SW620 and CACO2 cells constitutively express cell-surface u-PAR. (i) SW480, (ii) SW620 and (iii) CACO2 cells were analysed by FACScan analysis as described in Materials and Methods. Filled histograms represent isotype-matched mAb that served as a negative control; open histograms represent anti-u-PAR mAb. Shown are data from one representative experiment from three independent assays. (**B**) LPS enhances total cellular u-PAR expression. Following stimulation with various concentrations of LPS for 24 h, the u-PAR concentration in SW480 and SW620 cell lysates was measured by ELISA. Data are expressed as the mean±s.d. (ng per mg of protein) of six separate experiments conducted in triplicate. The statistical significance was compared with cells incubated with culture medium alone (^*^ for 0.1 *μ*g ml^−1^, *P*<0.05) and 0.1 *μ*g LPS (^**^P=<0.05 for 1 *μ*g LPS compared to 0.1 *μ*g LPS) and between SW480 and SW620 cells (^**^ for SW480 cells, *P*<0.05). (**C**) LPS enhances cell-surface u-PAR expression. Surface expression of u-PAR on SW480 (i) and SW620 (ii) cells was determined by FACScan analysis after stimulation with LPS for 24 h. Filled histograms represent 0 *μ*g ml^−1^ LPS+u-PAR mAb; open histograms represent 0.1 *μ*g ml^−1^ LPS+u-PAR mAb. Shown are data from one representative experiment with tabulated MFI (±s.e.m.) from three independent assays.

SW480, SW620 and CACO2 tumour cells constitutively expressed cell-surface u-PAR as confirmed by FACScan analysis (Figure 2A). Baseline total cellular u-PAR levels were significantly higher in primary SW480 cells than in metastatic SW620 cells (Figure 2B). LPS increased total cellular and surface u-PAR levels in a dose-dependent manner (Figure 2B and C).

### LPS increases tumour cell adhesion and invasion

**Figure 3:**
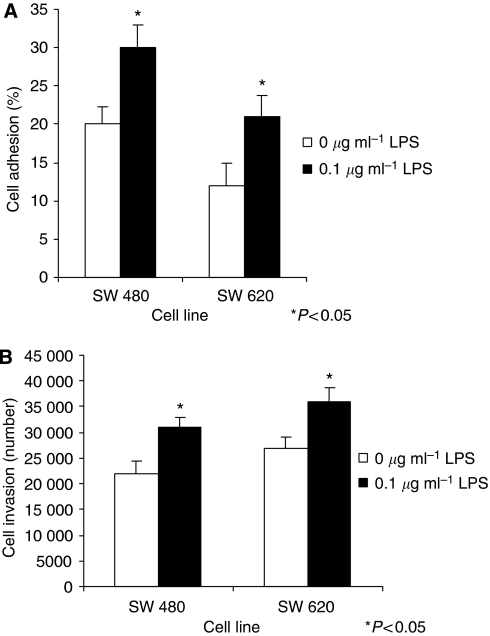
(**A**) LPS enhances SW480 and SW620 attachment to the extracellular matrix (ECM) protein vitronectin. After the cells were incubated with culture medium, 0.1 or 1 *μ*g ml^−1^ LPS for 24 h, the adherence of SW480 and SW620 cells to vitronectin was assessed as described in Materials and Methods. Data are expressed as the mean±s.d. from four separate experiments conducted in triplicate. Statistical significance was compared with the cells incubated with culture medium alone (^*^ for 0.1 *μ*g ml^−1^, *P*<0.05). (**B**) LPS enhances SW480 and SW620 ECM invasion. After the cells were incubated with culture medium, 0.1 or 1 *μ*g ml^−1^ LPS for 24 h, the ECM invasion of SW480 and SW620 cells was assessed as described in Materials and Methods. Data are expressed as the mean±s.d. from four separate experiments conducted in triplicate. Statistical significance was compared with the cells incubated with culture medium alone (^*^ for 0.1 *μ*g ml^−1^, *P*<0.05).

Different constitutive adhesion to the ECM protein vitronectin and *in vitro* invasion through ECM were observed between naive SW480 and SW620 cells (Figure 3A and B). Non-specific poly-D-lysine binding was similar in both cell lines and for LPS-stimulated and unstimulated cells (data not shown). LPS significantly increased tumour cell vitronectin adhesion (Figure 3A). Both cell lines demonstrated a significant 38% increase in vitronectin adhesion when stimulated with 0.1 *μ*g ml^−1^ LPS (*P*<0.05 compared to cells treated with culture medium alone). *In vitro* tumour cell invasion was also enhanced by approximately 43% in SW480 and SW620 cells treated with 0.1 *μ*g ml^−1^ LPS *versus* culture medium alone (*P*<0.05; Figure 3B).

### Inhibition of u-PAR and u-PA attenuates LPS-mediated tumour cell adhesion and invasion

**Figure 4:**
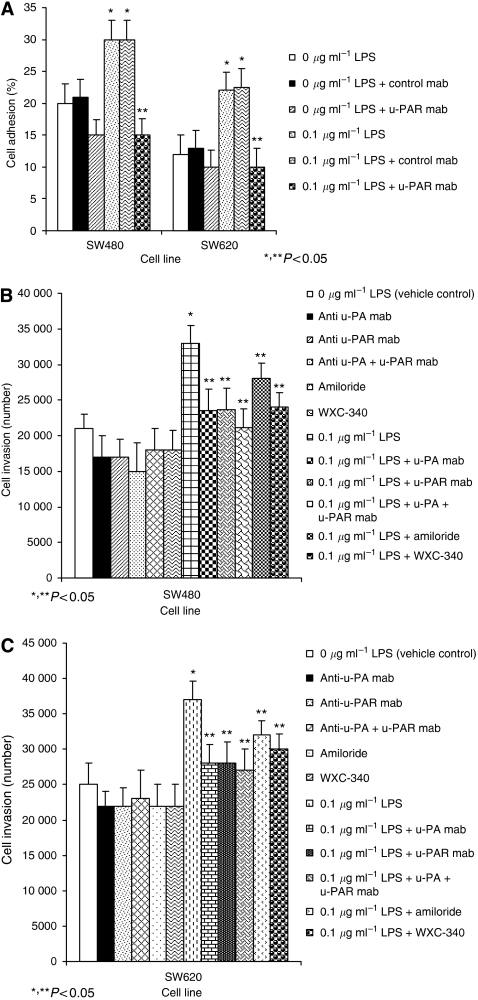
Effect of u-PA and u-PAR blockade on LPS-induced tumour cell adhesion and invasion. SW480 and SW620 cells were incubated with either isotype-matched control mAb (2.5 *μ*g ml^−1^) or u-PAR function-blocking mAb (2.5 *μ*g ml^−1^) or the selective u-PA inhibitors amiloride (10 *μ*g ml^−1^) and WXC-340 (1 *μ*g ml^−1^) for 1 h before 0.1 *μ*g ml^−1^ LPS stimulation. Tumour cell attachment to vitronectin (**A**) and ECM invasion (**B** and **C**) were assessed as described in Materials and Methods. Results are expressed as the mean±s.d. from three separate experiments conducted in triplicate. Statistical significance was compared with cells incubated with either culture medium plus control mAb (^*^P<0.05) or LPS plus control mAb (^**^P<0.05).

SW480 and SW620 cells pre-incubated with the u-PAR function-blocking mAb for 1 h before LPS stimulation failed to demonstrate enhanced vitronectin adhesion (*P*<0.05 compared to cells treated with LPS or LPS plus isotype mAb control) (Figure 4A). Pre-treatment with either u-PA (20 *μ*g ml^−1^) or u-PAR (20 *μ*g ml^−1^) function-blocking mAbs or the selective u-PA inhibitors, amiloride (10 mg ml^−1^) and WXC-340 (0.3 mg ml^−1^) partially impaired LPS-enhanced tumour cell invasion (*P*<0.05 compared to cells treated with LPS or LPS plus isotype mAb control) (Figure 4B and C). Combined u-PA and u-PAR inhibition further impaired both basal and LPS-stimulated tumour cell invasion in an additive manner (*P*<0.05 compared to cells treated with LPS or LPS plus u-PA, u-PAR or isotype-control mAb alone) (Figure 4B and C).

### SW480 and SW620 cells express TLR-4 whereas TLR-4 inhibition attenuates LPS-mediated activation of the u-PA and u-PAR system, tumour cell adhesion and invasion

**Figure 5:**
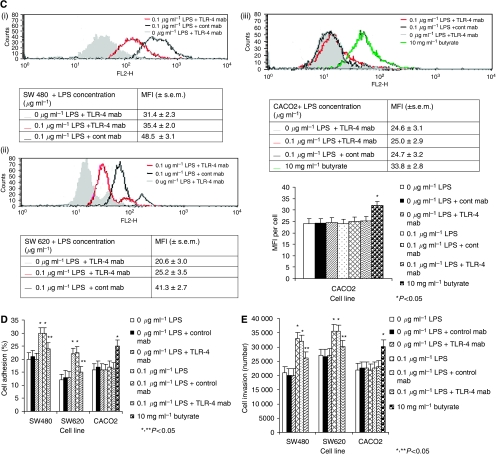
(**A**) SW480, SW620 and CACO2 constitutively express cell-surface TLR-4. (i) SW480, (ii) SW620, (iii) CACO2 and (iv) THP-1 (positive control) cells were analysed by flow cytometry using direct immunofluorescent staining as described in Materials and Methods. Filled histograms representing isotype-matched mAbs served as a negative control; open histograms represent anti-TLR-4 mAb. Shown are data from one representative experiment from three independent assays. (**B**, i–iii) Inhibition or deficiency of TLR-4 reduces LPS-enhanced u-PA activity. Following pre-incubation with 20 mg ml^−1^ anti-TLR-4 function-blocking antibody or matched isotype control, cell supernatant u-PA activity were analysed by colorimetric analysis for SW480 (i), SW620 (ii) and CACO2 (iii) cell lines. Data are expressed as the mean±s.d. from six separate experiments conducted in triplicate. Statistical significance was compared with cells incubated in either culture medium alone (^*^P<0.05) or 0.1 *μ*g ml^−1^ LPS (^**^P<0.05). (**C**, i–iii) Inhibition or deficiency of TLR-4 reduces LPS-enhanced surface u-PAR expression. Following pre-incubation with 20 mg ml^−1^ anti-TLR-4 function-blocking antibody or matched isotype control, surface u-PAR expression was analysed by flow cytometry for SW480 (i), SW620 (ii) and CACO2 (iii) cell lines. Filled histograms represent 0 *μ*g ml^−1^ LPS+TLR-4 mAb; open histograms: red line represents 0.1 *μ*g ml^−1^ LPS+TLR-4 mAb, black line 0.1 *μ*g ml^−1^ LPS+cont mAb and blue line 10 mg ml^−1^ butyrate. Shown are data from one representative experiment with tabulated MFI (±s.e.m.) from six independent assays. Statistical significance was compared with cells incubated in either culture medium alone (^*^P<0.05) or 0.1 *μ*g ml^−1^ LPS (^**^P<0.05). (**D**) Inhibition or deficiency of TLR-4 reduces LPS-enhanced tumour cell vitronectin adhesion. Following pre-incubation with 20 *μ*g ml^−1^ anti-TLR-4 function-blocking antibody or matched isotype control, tumour cells were stimulated with 0.1 *μ*g ml^−1^ of LPS for 24 h and vitronectin adhesion assessed as described in Materials and Methods. Results are expressed as the mean±s.d. from four separate experiments, conducted in triplicate. Statistical significance was compared with cells incubated in either culture medium alone (^*^P<0.05) or 0.1 *μ*g ml^−1^ LPS (^**^P<0.05). (**E**) Inhibition or deficiency of TLR-4 leads to reduced LPS-stimulated tumour cell extracellular matrix (ECM) invasion. Following pre-incubation with 20 *μ*g ml^−1^ anti-TLR-4 function-blocking antibody or matched isotype control, tumour cells were stimulated with 0.1 *μ*g ml^−1^ LPS for 24 h ECM invasion assessed as described in Materials and Methods. Results are expressed as the mean±s.d. from four separate experiments, conducted in triplicate. Statistical significance was compared with cells incubated in either culture medium alone (^*^P<0.05) or 0.1 *μ*g ml^−1^ LPS (^**^P<0.05).

Both SW480 and SW620 cells showed low constitutive expression of TLR-4 on the cell surface whereas CACO2 cells lacked TLR-4 surface expression, as confirmed by FACScan analysis (Figure 5A). CACO2 cells failed to demonstrate increased u-PA or u-PAR expression after stimulation with varying concentrations of LPS but did when stimulated with butyrate (Figure 5B and C). Pre-treatment of SW480 and SW620 cells with a TLR-4-blocking Ab (25 *μ*g ml^−1^ HTA-125) for 1 h before LPS stimulation significantly reduced both u-PA and u-PAR expression and u-PA activity (*P*<0.05 compared to cells stimulated with LPS alone or LPS plus isotype Ab control) (Figure 5B and C). CACO2 cells failed to demonstrate increased tumour cell vitronectin adhesion (Figure 5D) and *in vitro* tumour cell invasion (Figure 5E) in response to LPS stimulation. TLR-4 blockade also significantly reduced LPS-dependent tumour cell vitronectin adhesion (Figure 5D) and *in vitro* tumour cell invasion for SW480 and SW620 cell lines (Figure 5E).

### LPS increases NF-*κ*B activation whereas NF-*κ*B inhibition attenuates LPS-mediated activation of the u-PA and u-PAR system, tumour cell adhesion and invasion

**Figure 6:**
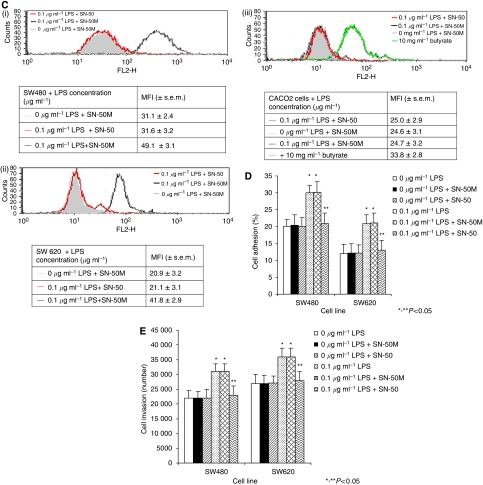
(**A**) LPS stimulation of SW480 and SW620 cells increased NF-*κ*B activity. SW480 (i), SW620 (ii) and CACO2 (iii) cell lines were stimulated with 0.1 *μ*g ml^−1^ for 30 min cell after incubation with TLR-4 mAb, control mAb or medium alone. Cell and nuclear lysates were obtained and NF-*κ*B activity assessed by ELISA as described in Materials and Methods. Data are expressed as the mean±s.d. of three separate experiments conducted in triplicate. Statistical significance was compared with cells incubated in either culture medium alone (^*^P<0.05) or 0.1 *μ*g ml^−1^ LPS (^**^P<0.05). (**B**, i–iii) NF-*κ*B inhibition impairs LPS-enhanced u-PA activity. Following pre-incubation with 100 mg ml^−1^ SN-50 or SN-50M and subsequent stimulation with 0.1 *μ*g ml^−1^ LPS, cell supernatant u-PA activity for SW480 (i), SW620 (ii) and CACO2 (iii) cells was analysed by colorimetric analysis as described in Materials and Methods. Data are expressed as the mean±s.d. of six separate experiments conducted in triplicate. Statistical significance was compared with cells incubated in either culture medium alone (^*^P<0.05) or 0.1 *μ*g ml^−1^ LPS (^**^P<0.05). (**C**) NF-*κ*B inhibition impairs LPS-enhanced u-PAR surface expression. Following pre-incubation with 100 mg ml^−1^ SN-50 or MSN-50 and subsequent stimulation with 0.1 *μ*g ml^−1^ LPS, surface u-PAR was analysed by flow cytometry as described in Materials and Methods for SW480 (i), SW620 (ii) and CACO2 (iii) cell lines. Filled histograms represent 0 *μ*g ml^−1^ LPS+SN-50M; open histograms: red lines represent 0.1 *μ*g ml^−1^ LPS+SN-50, black lines 0.1 *μ*g ml^−1^ LPS+SN-50M and green lines 10 mg ml^−1^ butyrate. Shown are data from one representative experiment with tabulated MFI (±s.e.m.) from six independent assays. Statistical significance was compared with cells incubated in either culture medium alone (^*^P<0.05) or 0.1 *μ*g ml^−1^ LPS (^**^P<0.05). (**D**) NF-*κ*B inhibition impairs LPS-enhanced tumour cell vitronectin adhesion. Following pre-incubation with 100 mg ml^−1^ SN-50 or SN-50M and subsequent stimulation with 0.1 *μ*g ml^−1^ LPS tumour cell attachment to vitronectin was assessed as described in Materials and Methods. Results are expressed as the mean±s.d. from four separate experiments, conducted in triplicate. Statistical significance was compared with cells incubated with culture medium alone (^*^P<0.05) or 0.1 *μ*g ml^−1^ LPS (^**^P<0.05). (**E**) NF-*κ*B inhibition impairs LPS-enhanced tumour cell ECM invasion. Following pre-incubation with 100 mg ml^−1^ SN-50 or SN-50M and subsequent stimulation with 0.1 *μ*g ml^−1^ LPS, tumour cell ECM invasion was assessed as described in Materials and Methods. Results are expressed as the mean±s.d. from four separate experiments, conducted in triplicate. Statistical significance was compared with cells incubated with culture medium alone (^*^P<0.05) or 0.1 *μ*g ml^−1^ LPS (^**^P<0.05).

Stimulation of SW480 and SW620 cells with 0.1 *μ*g ml^−1^ LPS for 30 min increases NF-*κ*B activity. This LPS-enhanced NF-*κ*B activity is attenuated by TLR-4 inhibition (Figure 6A). SN-50 is a cell-permeable peptide containing a hydrophobic N-terminal linked to the nuclear localisation sequence of NF-*κ*B p50, which prevents nuclear translocation of LPS and TNF-*α*-activated NF-*κ*B. SN-50M has two-peptide substitution and no measurable effect on NF-*κ*B activation in LPS-stimulated cells. Pre-treatment of SW480 and SW620 cells with 100 *μ*g ml^−1^ of SN-50, for 1 h before LPS stimulation, inhibited LPS-induced upregulation of u-PA activity (Figure 6B) and u-PAR expression (Figure 6C) compared to cells stimulated with LPS (*P*<0.05) or LPS plus SN-50M (*P*<0.05). Furthermore, SN-50 pre-treatment attenuated LPS-dependent tumour cell vitronectin adhesion (Figure 6D) and *in vitro* ECM invasion (Figure 6E) compared to cells stimulated with LPS (*P*<0.05) or LPS plus SN-50M (*P*<0.05).

### Protein synthesis inhibition impairs LPS-mediated activation of the u-PA and u-PAR system

Cycloheximide is a protein synthesis inhibitor that inhibits the initiation of new peptide chains and the elongation of nascent peptides on ribosomes by different mechanisms (Ennis and Lubin, 1964).

**Figure 7:**
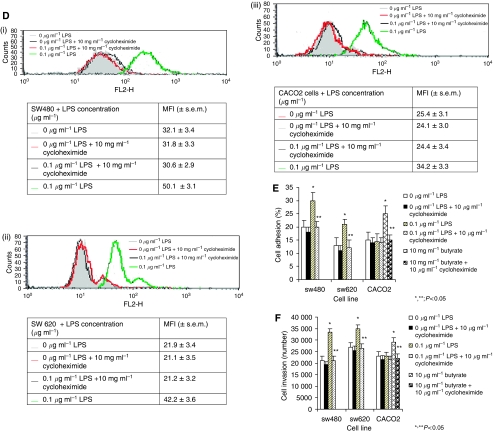
(**A**) Protein synthesis inhibition impairs LPS-enhanced cell supernatant u-PA concentration. Following co-incubation with 10 *μ*g ml^−1^ cycloheximide and 0.1 *μ*g ml^−1^ LPS, cell supernatant u-PA concentration for SW480 (i), SW620 (ii) and CACO2 (iii) cells was analysed by ELISA as described in Materials and Methods. Data are expressed as the mean±s.d. of six separate experiments conducted in triplicate. Statistical significance was compared with cells incubated in either culture medium alone (^*^P<0.05) or 0.1 *μ*g ml^−1^ LPS (^**^P<0.05). (**B**) Protein synthesis inhibition impairs LPS-enhanced u-PA activity. Following co-incubation with 10 *μ*g ml^−1^ cycloheximide and 0.1 *μ*g ml^−1^ LPS, cell supernatant u-PA activity for SW480 (i), SW620 (ii) and CACO2 (iii) cells was analysed by colorimetric analysis as described in Materials and Methods. Data are expressed as the mean±s.d. of six separate experiments conducted in triplicate. Statistical significance was compared with cells incubated in either culture medium alone (^*^P<0.05) or 0.1 *μ*g ml^−1^ LPS (^**^P<0.05). (**C**) Protein synthesis inhibition impairs LPS-enhanced total u-PAR expression. Following co-incubation with 10 *μ*g ml^−1^ cycloheximide and 0.1 *μ*g ml^−1^ LPS, total cellular u-PAR expression was determined by ELISA as described in Materials and Methods. Data are expressed as the mean±s.d. of six separate experiments conducted in triplicate. Statistical significance was compared with cells incubated in either culture medium alone (^*^P<0.05) or 0.1 *μ*g ml^−1^ LPS (^**^P<0.05). (**D**) Protein synthesis inhibition impairs LPS-enhanced surface u-PAR expression. Following co-incubation with 10 *μ*g ml^−1^ cycloheximide and 0.1 *μ*g ml^−1^ LPS, cell-surface u-PAR was analysed by flow cytometry as described in Materials and Methods for SW480 (i), SW620 (ii) and CACO2 (iii) cell lines. Filled histograms represent 0 *μ*g ml^−1^; open histograms: red lines represent 0 *μ*g ml^−1^ LPS+10 *μ*g ml^−1^ cycloheximide, black lines 0.1 *μ*g ml^−1^ LPS+10 *μ*g ml^−1^ cycloheximide and green lines 0.1 *μ*g ml^−1^ LPS. Shown are data from one representative experiment with tabulated MFI (±s.e.m.) from six independent assays. Statistical significance was compared with cells incubated in either culture medium alone (^*^P<0.05) or 0.1 *μ*g ml^−1^ LPS (^**^P<0.05). (**E**) Protein synthesis inhibition impairs LPS-enhanced tumour cell vitronectin adhesion. Following co-incubation with 10 *μ*g ml^−1^ cycloheximide and 0.1 *μ*g ml^−1^ LPS, tumour cell attachment to vitronectin was assessed as described in Materials and Methods. Results are expressed as the mean±s.d. from four separate experiments, conducted in triplicate. Statistical significance was compared with cells incubated with culture medium alone (^*^P<0.05) or 0.1 *μ*g ml^−1^ LPS (^**^P<0.05). (**F**) Protein synthesis inhibition impairs LPS-enhanced tumour cell ECM invasion. Following co-incubation with 10 *μ*g ml^−1^ cycloheximide and 0.1 *μ*g ml^−1^ LPS, tumour cell ECM invasion was assessed as described in Materials and Methods. Results are expressed as the mean±s.d. from four separate experiments, conducted in triplicate. Statistical significance was compared with cells incubated with culture medium alone (^*^P<0.05) or 0.1 *μ*g ml^−1^ LPS (^**^P<0.05).

Co-incubation of SW480 and SW620 cells with 10 *μ*g ml^−1^ of cycloheximide inhibited LPS-induced upregulation of u-PA (Figure 7A), u- PA activity (Figure 7B), total (Figure 7C) and surface u-PAR expression (Figure 7D) compared to cells stimulated with LPS alone (*P*<0.05). Furthermore cycloheximide attenuated LPS-dependent tumour cell vitronectin adhesion (Figure 7E) and *in vitro* ECM invasion (Figure 7F) compared to cells stimulated with LPS alone (*P*<0.05). This suggests that the stimulatory effect of LPS is at least partially mediated at the transcriptional level.

## Discussion

Bacterial endotoxin directly and indirectly facilitates cancer progression *in vitro* and *in vivo* through a multitude of complimentary mechanisms. Fundamental to this process is tumour cell invasion involving cell attachment to the subendothelial ECM and subsequent unidirectional cell migration coupled with local proteolysis induced by a number of enzymes, particularly MMPs and u-PA (Pollanen *et al*, 1988; Sidenius and Blasi, 2003). We have previously demonstrated that LPS enhances tumour cell invasion in colorectal tumour cells through a mechanism at least partially mediated by NF-*κ*B-dependent *β*1 integrin upregulation (Wang *et al*, 2003). However invasion is ultimately subservient in many cases to local pericellular proteolysis. SW480 and SW620 express low levels of MMPs, which were unaltered with LPS stimulation (data not shown). Therefore this study focused on the contribution of another proteolytic cascade, the u-PA system, to endotoxin-mediated accelerated tumour invasion.

The SW480 cell line was cultured from a primary rectal tumour and the SW620 cell line from a secondary hepatic metastases derived from the same patient. Interestingly, basal u-PA and u-PA activity levels were approximately 40% higher in metastatic SW620 cells compared to primary SW480 cells. This was mirrored in higher basal levels of *in vitro* invasion using the ECM invasion chamber. In contrast, baseline surface and total cellular expression of u-PAR was higher in SW480 cells when compared to SW620 cells, and therefore, adhesion to its specific ligand vitronectin was correspondingly higher in SW480 cells. Although both increased u-PA and u-PAR levels are associated with tumour metastases (Duffy, 2002), elevated u-PA expression may be important in development of the metastatic process in this cell system as acquisition of the metastatic phenotype appears to involve overexpression of u-PA and downregulation of u-PAR. Notwithstanding the differences in basal expression, the relative increase in u-PA protein concentration and activity, and u-PAR surface and total cellular expression with LPS stimulation were similar between both SW480 and SW620 cell lines. Tumour cell vitronectin adhesion and *in vitro* ECM invasion were likewise consistently amplified with LPS challenge.

Baseline and LPS-associated *in vitro* cellular invasion was partially repressed by u-PA and u-PAR inhibition. The selective u-PA inhibitor amiloride and novel chemotherapeutic compound WXC-340 also incompletely reduced LPS-enhanced invasion. This reduction occurred to the same extent in both SW480 and SW620 cells. Combined u-PAR and u-PA inhibition further impaired invasion in an additive manner. Such findings incriminate the u-PA system as a pre-eminent end effector mechanism in LPS-mediated enhanced tumour invasion. Constituents of this system could justifiably be targeted in the perioperative period. Indeed, the precursor of WXC-340, wx-uk1 has completed phase I trials in patients with metastatic solid tumours tumours (http://clinicaltrials.gov/ct2/results?term=wx-uk1).

LPS signalling involves TLR-4, a member of the highly conserved family of TLR proteins (Schumann *et al*, 1990; Perera *et al*, 2001). Both SW480 and SW620 cells express low but measurable surface levels of TLR-4 whereas CACO2 do not, reflecting the poor response of this cell line to LPS stimulation. Furthermore, LPS-mediated u-PA and u-PAR upregulation and enhanced tumour cell adhesion and invasion was abrogated by TLR-4 blockade using a functional TLR-4-blocking mAb.

On stimulation of TLR-4 with LPS, I*κ*B-*α* is phosphorylated by I*κ*B-*α* kinase and thus phosphor-I*κ*B-*α* is transiently expressed in the cytoplasm concomitant with a similarly transient decrease in cytoplasmic I*κ*B-*α* (Aggarwal, 2004). In this study blockade of NF-*κ*B activation by SN-50, a synthetic peptide that impedes NF-*κ*B signalling by inhibition of nuclear translocation of NF-*κ*B, abrogated LPS-induced upregulation of the u-PA system and attenuated tumour cell adhesion and invasion, indicating that NF-*κ*B activation is a prerequisite not only for the transduction of LPS signals in tumour cells, but also for the enhanced tumour cell metastatic ability induced by LPS stimulation. Protein synthesis seems fundamental to this LPS-induced upregulation of the u-PA system and enhanced tumour cell adhesion and invasion (Figure 7A–F).

LPS antagonism is another potential perioperative therapy, particularly attractive as it would not impair putatively important TLR-4 or NF-*κ*B function in normal tissue.

Thus stimulation of tumour cell TLR-4 and subsequent NF-*κ*B activation by systemic exposure to LPS in the perioperative period may accentuate tumour cell adhesion and invasion by a variety of mechanisms including activation of the u-PA and integrin systems.

**Figure 8:**
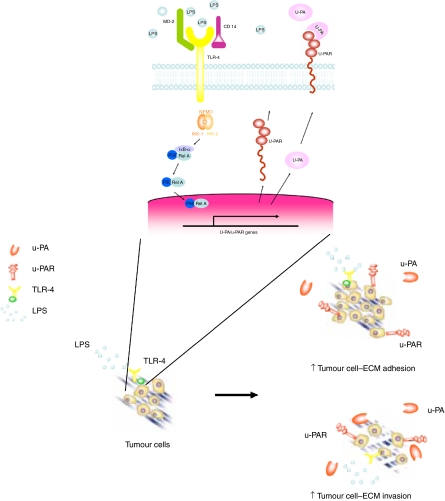
Binding of LPS to TLR-4 leads to NF-*κ*B activation, increased u-PA activity and u-PAR expression and ultimately enhanced tumour cell vitronectin adhesion and tumour cell extracellular matrix invasion.

In conclusion, bacterial endotoxin directly promotes tumour cell adhesion and invasion through the upregulation of u-PA and u-PAR mediated by TLR-4-dependent activation of NF-*κ*B (Figure 8). These findings provide further evidence for the involvement of bacterial products in surgery-associated accelerated growth in metastatic disease and justify either selective or collective targeting of LPS, TLR-4 and the u-PA system in the perioperative period to attenuate surgery-induced accelerated metastatic tumour growth.
